# Revolutionizing anti-tumor therapy: unleashing the potential of B cell-derived exosomes

**DOI:** 10.3389/fimmu.2023.1188760

**Published:** 2023-06-05

**Authors:** Jingwen Xiong, Hao Chi, Guanhu Yang, Songyun Zhao, Jing Zhang, Lisa Jia Tran, Zhijia Xia, Fang Yang, Gang Tian

**Affiliations:** ^1^ Department of Sports Rehabilitation, Southwest Medical University, Luzhou, China; ^2^ Clinical Medical College, Southwest Medical University, Luzhou, China; ^3^ Department of Specialty Medicine, Ohio University, Athens, OH, United States; ^4^ Department of Neurosurgery, Wuxi People’s Hospital Affiliated to Nanjing Medical University, Wuxi, China; ^5^ Division of Basic Biomedical Sciences, The University of South Dakota Sanford School of Medicine, Vermillion, SD, United States; ^6^ Department of General, Visceral, and Transplant Surgery, Ludwig-Maximilians-University Munich, Munich, Germany; ^7^ Department of Ophthalmology, Charité – Universitätsmedizin Berlin, Berlin, Germany; ^8^ Department of Laboratory Medicine, The Affiliated Hospital of Southwest Medical University, Luzhou, China

**Keywords:** B cell, B cell-derived exosome, therapy, TME, anti-tumor

## Abstract

B cells occupy a vital role in the functioning of the immune system, working in tandem with T cells to either suppress or promote tumor growth within the tumor microenvironment(TME). In addition to direct cell-to-cell communication, B cells and other cells release exosomes, small membrane vesicles ranging in size from 30-150 nm, that facilitate intercellular signaling. Exosome research is an important development in cancer research, as they have been shown to carry various molecules such as major histocompatibility complex(MHC) molecules and integrins, which regulate the TME. Given the close association between TME and cancer development, targeting substances within the TME has emerged as a promising strategy for cancer therapy. This review aims to present a comprehensive overview of the contributions made by B cells and exosomes to the tumor microenvironment (TME). Additionally, we delve into the potential role of B cell-derived exosomes in the progression of cancer.

## 1 Introduction

Cancer remains a significant global cause of mortality (1). Historically, cancer research solely emphasized studying cancer cells. However, with the introduction of the “seed and soil” concept (2), researchers have redirected their focus towards investigating the development of cancer cells within TME. The TME fosters cancer cell growth and progression through its complex composition of ECM, neuroendocrine cells, immune cells, stromal cells, fibroblasts, and lymphatic networks (3, 4). Immune cells play a significant role in the survival of tumors, as cancer cell metabolites and secretions from specific TME cells can influence the activation, proliferation, differentiation, and function of immune cells (5).

Reports suggest that extracellular vesicles (EVs), particularly exosomes, hold significant potential as a cancer treatment (6). Standard chemotherapy methods may cause harm to normal cells, resulting in detrimental side effects (7, 8). In contrast, Targeted therapy has become a more appealing approach to combatting tumors as it offers greater specificity and can spare adjacent healthy tissues (9). The idea of utilizing exosomes for targeted therapy is intriguing, given that almost all cells produce exosomes (10). Bioengineered exosomes have garnered considerable attention due to their exceptional stability, extensive tissue penetrability, potent targeting capability, and precise drug modulatory properties (11). Although there are no standardized protocols for exosome isolation and purification (12), their potential role in cancer therapy warrants further investigation into their biological functions.

B cells are present in both secondary lymphoid organs (SLOs) and tertiary lymphoid structures(TLSs). TLSs, which represent lymphoid neogenesis sites that occur in most solid tumors (13), primarily consist of B cell follicles and T cell zones, along with mature dendritic cells (DCs) (14, 15). Within these lymphoid structures, there is a rich signaling crosstalk between B cell-derived exosomes and other cells.

## 2 B cells and the tumor microenvironment

In the past decade, immune cells have gained significant attention in TME research due to their capacity to regulate tumor growth (16). Despite incomplete comprehension of the role of B cells in cancer research, their heterogeneity has been demonstrated as indispensable in the TME (17). B cells are also involved in the formation of TLSs, which has been instrumental in advancing cancer research (18).

### 2.1 Immunomodulatory functions of B cells

B cells are a component of the adaptive immune system that can differentiate into various subsets when subjected to diverse stimuli and stress conditions (19). The multifaceted nature of B cell subsets, which is primarily due to the absence of a precise definition of their transcription factors, makes comprehending their functions a difficult and complicated process (17).

B cells assume a pivotal role in antibody production and antigen presentation to facilitate effective immune responses among other immune cells (20, 21). After antigen recognition, B cell activation occurs through activation of B cell receptors (BCRs) and Toll-like receptors present on the surface of them. Subsequently, activated B cells differentiate into plasma cells through two distinct pathways. In the first pathway, plasma cells differentiate outside the lymphoid follicle, exhibit lower affinity for antigens, and have shorter lifespans. In the second pathway, B cells migrate into the follicle and establish a germinal center (GC) that differentiates into long-lived plasma cells and memory B cells (22–24). Plasma cells produce IgM antibodies that contribute to humoral immune responses. Moreover, B-cell-associated immunoglobulins, including IgG, IgE, and IgA, have been extensively reviewed in the literature with respect to their subclasses (21). IL-10 promotes plasma cell production through CD40 activation and is superior to IL-4 (25). Recent studies have found that tumour-associated neutrophils in TME rely on TNF-α to recruit B cells and regulate B cell differentiation into plasma cells *via* the BAFF pathway (26).

Memory B cells exhibit signaling molecules, such as MHC and co-stimulatory molecules, along with cytokines (IL-6, TNF, GM-CSF) on their surface, which stimulate T cells, thereby amplifying the immune response (24). Upon a second exposure to antigens, memory B cells produce high-quality antibodies (27).

B regulatory (Breg) cells are distinct in that their primary function is to suppress the immune system (28). Breg subsets containing IL-10 and IL-35 have the ability to suppress effector T cells (both CD4 and CD8), NK cells, and neutrophils (29). Additionally, Bregs regulate levels of extracellular metabolites such as ATP, ADP, AMP, and adenosine in TME, resulting in the suppression of T and B cell proliferation, forming a complex network (30, 31).

Tumor-infiltrating B cells (TIBs) participate in the development of TLSs, which stimulate an active anti-tumor response *via* antigen presentation (32, 33). Activated TIBs release enzymes or receptors to kill cancerous cells, leading to their lysis (34). B cells secrete angiogenic factors to stimulate the activation of STAT3 and facilitate angiogenesis (35). In prostate cancer, TIBs may produce lymphotoxin (LT), and high levels of LT can lead to CR-CAP and adversely affect treatment outcomes (36).

### 2.2 B cells in cancer therapy

Surface markers CD19, CD20, and CD37 are expressed at varying levels by B cells during development. Although studies indicate that targeting these molecules therapeutically holds promise for B-cell cancers (37), their potential efficacy against other cancer types remains largely unexplored. Mediation by CD19 increases antigen presentation by B cells, consequently improving the T cell response (38). Attracting B cells, CXCL13 functions as a chemokine (36, 39). Tumor-induced Bregs (tBregs) serve as a marker of tumor persistence (40). CD20 is expressed on both anti-tumor B cells and tBregs, and targeting CD20 B cells with CXCL13-coupled CpG-ODN can enrich and inactivate tBregs, thereby controlling tumor immune escape (41). This approach minimizes the potential side effects of B-cell depletion methods. In bladder cancer, CXCL13 expression can be used as a surrogate marker for tumor TLSs and correlates with the response to immune checkpoint inhibitors (ICIs) in patients (42). Additionally, in a subset of patients with soft tissue sarcoma, B-cell-enriched TLSs was associated with a better response to anti-PD-1 blockade therapy and increased survival rates (43).

However, the inhibitory mechanisms of tumors mediated by TLSs and B cells in ICI therapy remain poorly understood (44). It is noteworthy that within GCs, B cells are activated to produce antibodies, while Bregs and Tregs produce cytokines such as IL-10, IL-35, and TGF-β to suppress T cell function (18). Furthermore, chemotherapy (45) and vaccination (46) have also demonstrated associations with TLSs.

B cell-enriched TLSs have been found to exhibit tumor-suppressing properties (47). Infiltration of CD19/CD20 B cells has now emerged as a promising target for immunotherapy of hepatocellular carcinoma (48). Thus, combining immunotherapies such as cancer vaccines, cytokine therapies, and immune checkpoint inhibitors (ICIs) with strategies targeting B cells and TLSs may represent promising new anti-tumor approaches.

## 3 Exosomes and tumor microenvironment

**Figure 1:**
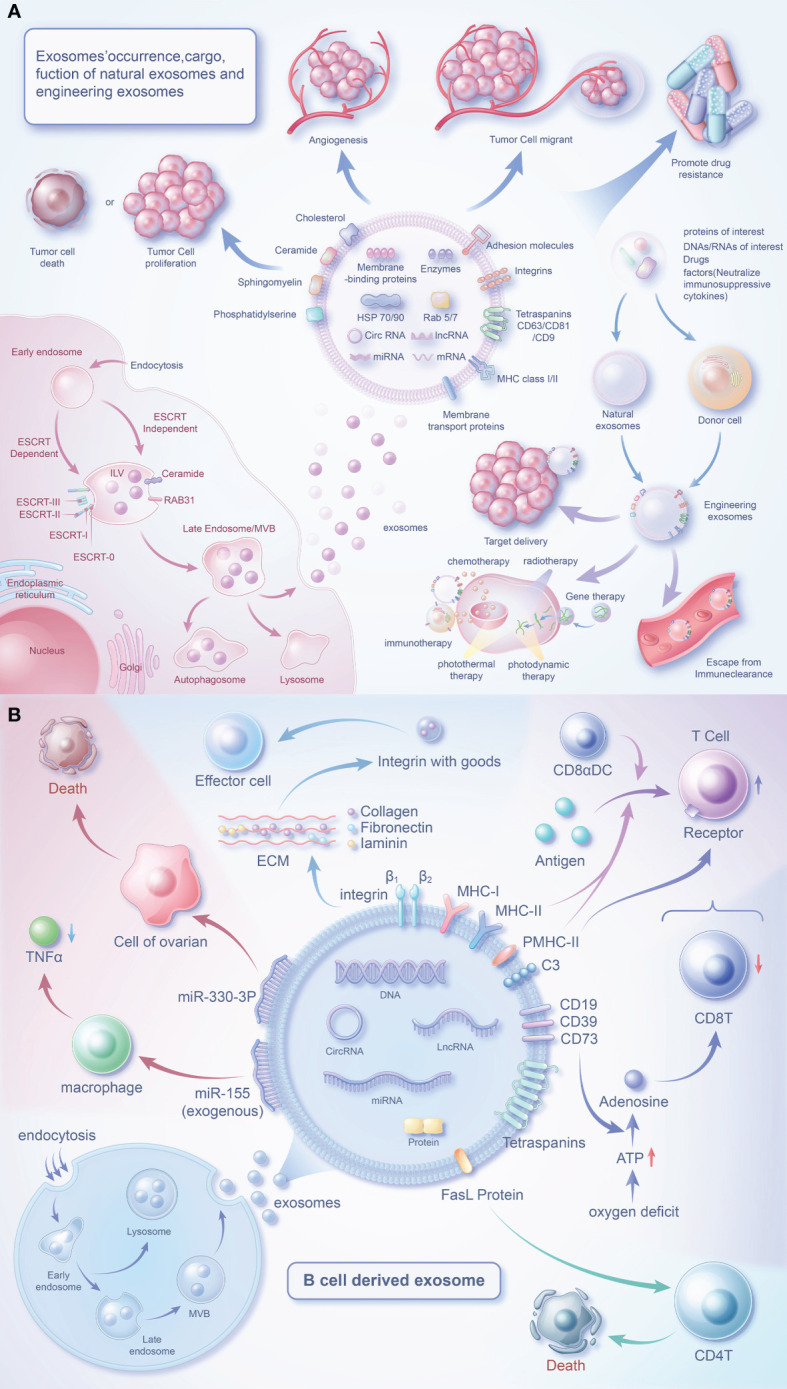
Biology of exosomes and the role of their cargo in the tumor microenvironment. **(A)** Exosomes are produced in dependence on the ESCRT mechanism or a RAB31-mediated pathway independent of the ESCRT mechanism. Exosomes carry a variety of proteins and effector molecules that can determine the direction of tumour development and tumour metastasis, promote tumour angiogenesis and participate in tumour drug resistance.Engineered exosomes carry cargoes of interest that also have multiple roles closely related to tumours. **(B)** B cells release exosomes through endocytosis of the plasma membrane, the formation of early endosomes and late endosomes, and fusion of polyvesicular bodies with the plasma membrane. In addition to MHC protein and quadruple transmembrane protein, the surface of B cell-derived exosomes contains special FasL proteins, integrins, C3, CD19, CD39, CD73, etc., which can regulate immune cells (T cells) or affect the survival of tumor cells through special factors.

Exosomes facilitate substance transfer between cells, activating signaling pathways (49). The secretion of exosomes by B cells is among the most important ways in which they influence the TME. Additionally, exosomes secreted by other cells and cancer cells also play pivotal roles (50, 51). Therefore, it can be speculated that exosomes hold great potential for advancing the exploration of new therapeutic pathways in cancer (**Figure 1A**).

### 3.1 The biological characteristics and functions of exosomes

Exosomes, a type of extracellular vesicles, have been extensively studied (52–55). Exosomes are generated through the intracellular process of inward budding of multivesicular bodies, followed by their subsequent release into the extracellular space through fusion events with the plasma membrane (56). The secretion of exosomes is regulated by the RAB family (57, 58). Secretion of exosomes can be divided into an ESCRT mechanism and a RAB31-mediated mechanism independent of ESCRT (59). Despite being previously viewed as waste materials, exosomes are now acknowledged as vital components of the intercellular communication network (60). Exosomes, containing biologically active substances can be found in bodily fluids and are discharged by originating cells (54, 61). Exosomes are structurally more stable than parent cells due to their higher concentration of lipid components (62). CD81 and CD63 are common exosome markers, and along with CD82 and CD37, are highly abundant transmembrane proteins in exosomes. Exosomes also carry a diverse range of membrane signaling proteins (63). These signaling molecules can be delivered to target cells by exosomes (64), making them an integral component of TME communication.

As researchers continue to investigate exosomes, their overall functions have become increasingly evident. In a normal physiological context, exosomes contribute to the maintenance of immune response, cell proliferation, maturation, and homeostasis (65). Exosomes have been identified to play crucial roles in various processes within TME, including but not limited to immune regulation, promotion of cancer cell proliferation, metastasis, drug resistance, and angiogenesis (66, 67).

Exosomes can facilitate crosstalk between B cells and other cells, making them a potent target for cancer treatment (**Table 1**).

**Table 1:** Exosome crosstalk between B cells and other cells.

Other cell-derived exosomes act on B cells						
Tumor type	Source	In vivo/in vitro	Action type/specific axis	Effect	Effects on tumors	Ref.
——	DC	*In vivo*	Stimulate B cell proliferation	CD8 + T cell response was induced	Kill cancer cells	(68)
——	Mature DC	*In vivo*	Binds to B cell receptors	Induces T cell proliferation	——	(69)
——	T cells	*In vivo*	——	Promote B cell proliferation and differentiation	——	(70)
——	Mesenchymal cells	——	TGF-β1 is produced	Regulates B cell proliferation and survival	——	(71)
HCC	Tumor cells	*In vitro*	HMGB1-TLR2/4-MAPK	Induction of B cells to become TIM-1Breg, impairing CD 8T cell function	Promote HCC progression	(72)
HNSCC	plasma	*In vitro*	——	Inhibits the proliferation, viability and function of B cells	Helps immune escape	(73)
B-cell-derived exosomes act on other cells						
Tumor type	Target cells	*In vivo*/*in vitro*	Action type/specific axis	Effect	Effects on tumors	Ref.
Ovarian cancer	cancer cell	*In vitro*	miR-330-3p/JAM2 axis	Inducing mesenchymal procedures	Promote the growth of cancer cells	(74)
——	T cell	*In vivo*	Rab27a expression is up-regulated	Impaired CD8T cell response	Promote tumor survival	(75)
——	T cell	*In vivo*	C3 fragments are deposited on the surface of the cell membrane	Enhance T cell response, conducive to the development of immune response	Inhibits tumors	(76)
——	Macrophages	*In vivo*	Enhances lipopolysaccharide stimulation	Reduces the release of TNFα	Promote tumor survival	(77)

### 3.2 application of exosomes in cancer therapy

Exosomes are widely recognized as significant biomarkers for cancer diagnosis, prediction, and monitoring (78). Due to their stability and prevalence in circulation, they serve as valuable indicators for liquid biopsy techniques (79), enabling more accurate and less invasive treatments (80). Considering the active cross-talk of exosomes between immune and cancer cells, they can be utilized for immunotherapy to promote (81, 82) or suppress (83, 84) tumor proliferation. Additionally, cancer vaccines based on exosomes have also been developed (85).

Compared to other types of extracellular vesicles (microvesicles, membrane particles, apoptotic bodies), the majority of the biological components, lipids, and proteins of exosomes are relatively clear (86). In comparison to synthetic nanovesicles (polymeric nanoparticles, liposomes, and solid lipid nanoparticles), exosomes possess more membrane proteins, stronger biocompatibility, and a longer circulation half-life naturally (87). They can easily pass through the plasma membrane, blood, and blood-brain barrier (88) to infiltrate tumor tissues (89), which allows for precise and targeted therapeutic interventions (90). Consequently, emerging research is focused on the development and design of bioengineered exosomes.

Bioengineered exosomes can be designed to target specific cells, provide therapeutic cargo, and regulate the immune system (11).

Certain surface-adhesive proteins and carrier ligands allow exosomes to attach to target cell surfaces and efficiently deliver them into the cells (91). Exosomes can efficiently deliver drugs like paclitaxel (92), transport siRNA (93, 94). But the presence of the blood-brain barrier (BBB) limits the access of small molecule drugs to glioma tissue (95). Exosomes derived from brain endothelial cells can transport drugs to BBB, which may become a breakthrough for the treatment of brain cancer (96).

Immune suppressive factors can inhibit the function of immune cells (97–100). By transporting cargo that neutralizes these immune suppressive factors, exosomes can enhance the body’s anti-tumor immune response and improve the efficacy of cancer immunotherapy. An investigation has demonstrated that the T-cell suppressive effects of exosomes can be mitigated by the administration of anti-TGF-β (101). Franz L and colleagues conducted a PD-1 blockade experiment using EV secreted by glioblastoma, which almost reactivated T cells and suppressed tumor progression (102). In the HNSCC microenvironment, the T-cell inhibitory effect of circulating PD-L1 exosomes can be blocked by PD-1 antibodies (103). However, the interference of molecules carried by exosomes in immunotherapy may be related to mechanisms of drug resistance (104, 105).

## 4 B-cell-derived exosomes and the tumor microenvironment

The investigation of B cell-derived exosomes initiated with the initial characterization of these vesicles in 1996 (106). Through their surface effector molecules, these exosomes can signal to various cells to regulate the TME (**Figure 1B**).

### 4.1 The biological components of exosomes released by B cells

Mass spectrometry analysis has uncovered a wealth of constituents in exosomes originating from B cells, including MHC-I, MHC-II, CD20, CD45, and BCR complexes (comprising surface Ig, CD19, and Tetraspanins), as well as chaperones such as heat shock protein 70 and 90, integrins and other proteins (107–109). Furthermore, these exosomes also express cholesterol, sphingomyelin, and ganglioside GM3 (107). Addition of macrophage-derived exosomes to naive monocytes induces cell differentiation (110), however this mechanism is unclear in B cells and B cell exosomes. When B cells are activated, they release a greater amount of exosomes (111, 112), which carry more effector molecules (77, 108, 109). CD45 is a key positive regulator of the BCR-mediated signalling pathway (113, 114) and, interestingly, is absent from T-cell-derived exosomes (115, 116). Triggering the classical NF-κB pathway *via* downstream of BCR is able to increase HLA expression in B-cell exosomes (111). However, these two regulatory pathways may be linked and need to be explored in further experimental studies.

### 4.2 Mechanism of involvement of components of B cell-derived exosomes in the tumour process

#### 4.2.1 Proteins

MHC-II molecules in exosomes are predominantly located in SLOs and are expressed exclusively by professional APCs, including B cells (117). The presence of CD20, CD81, and HSC70 is associated with MHC-II on the plasma membrane, which promotes antigen presentation and T cell activation (118). CD20 can form complexes with both MHC-II and CD40 (119), while the R21 monoclonal antibody selectively recognizes CD20 on B cells. Additionally, R21 mAb can induce lfa-1-dependent cell adhesion but inhibits MHC-II-mediated lfa-1-dependent cell adhesion. It’s worth noting that CD40 may interfere with mAb binding to CD20 by competitively binding with it (119).

It is not surprising that exosomes are present in TLSs, as B cells are abundant in these structures (120). CD20 can form complexes with BCR to participate in signal transduction (121), ultimately initiating immune responses. Additionally, CD20 can activate B cells through calcium channels (122), and the resulting stimulated calcium channels may induce the release of a large number of exosomes (108). Some of the mechanisms of CD20 are not yet clear (37). The augmented abundance of CD20-positive B lymphocytes in sentinel lymph nodes is indicative of a favorable prognosis in cases of breast cancer (123). CD20 accumulates at the tumour-liver border in patients with colorectal cancer (124), where it may act as a prognostic marker for the tumour.

These indications suggest that these complexes may be crucial factors in MHC-II or antigen-mediated B cell responses.

B cell-derived exosomes express MHC-II and form a complex (PMHC-II) with peptide antigens. The notion that B-cell exosomal exosomes may transport PMHC-II, proposed more than 20 years ago (106), is confirmed. Exosomes released by most primary B cells express PMHC-II. When antigen-loaded B cells meet specific T cells, B cell activation stimulates exosome release, while stimulating pMHC-II to escape intracellular degradation. pMHC-II interacts with the TCR and activates naive CD4 T cells to initiate an immune response (125). However, exosomes from mature DCs only activate naïve T cells as inefficient APCs (126).

#### 4.2.2 ncRNAs

B-cell exosomes have emerged as viable candidates for the delivery of miRNA-155. When miRNA-155 inhibitor-loaded exosomes were administered to miRNA-155 knockout mice, a notable reduction in TNF-α production was observed in mouse RAW macrophages (77). After treatment with rituximab, a downregulation of miR-155 levels in exosomes was observed (127). This study highlights the potential of B cell-derived exosomes as therapeutic vehicles.

MiR-330-3p has been identified as a key factor in tumor progression. It targets TPX2, which negatively regulates TPX2 expression to inhibit melanoma cell proliferation (128). In addition, miR-330-3p from plasma cell-derived exosomes is a critical regulator of ovarian cancer stroma and promotes tumor metastasis through the JAM2 pathway (74). TPX2 (129) and JAM2 (130, 131) have been described as associated with cancer progression. However, the ability of miR-330-3p to target these proteins may suggest a potentially important role for our B-cell exosome miR-330-3p.

### 4.3 B cell-derived exosomes are taken up by other cells

#### 4.3.1 Follicular dendritic cells

*In vitro* isolated B cell-derived exosomes specifically bind follicular dendritic cells (FDC) (132). The fate of B cells in GC depends on the adhesion of the VCAM-1 pathway and is associated with FDC (133). More importantly, FDC themselves do not express MHC-II, but rather pick up peptide-loaded MHC-II on the surface of B cells (134). Exosome binding to FDC may be through interaction with VCAM-1, which then stimulates T helper cells. Additionally, tumor-associated FDCs express CXCL13, which effectively recruits lymphocytes (135). We postulate that in tumor tissues, FDCs may attract B cells, which then bind to the released exosomes, leading to antigen presentation. Therefore, FDCs may represent a physiological target for B cell-derived exosomes (132).

#### 4.3.2 Fibroblasts

Compared to FDCs, fibroblasts exhibit a limited expression of leukocyte adhesion molecules on their surface (132). Treatment of fibroblasts with TNF-α induces the upregulation of ICAM-1 expression on their surface, thereby enhancing the adhesion of B cell-derived exosomes to fibroblast surfaces (136). TNF-α may be a key factor in inducing exosomes to be adsorbed.

#### 4.3.3 Macrophages

In the subcapsular sinus of the lymph node, B-cell-derived exosomes are captured by CD169 macrophages, which then penetrate deep into the paracortex (137). CD169+ cells are present within B-cell follicles (138) and the T-B cell zone boundary of the GC (139). The presentation of antigens by CD169+ cells to CD8 T cells and/or B cells causes them to being activated (140). Activated B cells may release more exosomes. Exosome-induced CTL responses (141) are enhanced in CD169+ mice in cooperation with T and B cells, suggesting that exosomes enter lymphoid organs possibly to reduce the immune response to autoantigens (137).

## 5 Discussion

In recent years, exosomes have gained increasing attention as important mediators of intercellular communication. While many studies have focused on cancer cell-derived exosomes for diagnosis, prognostic testing, and drug delivery, less is known about exosomes derived from B cells. Fortunately, there are still some relevant studies targeting B-cell-derived exosomes, as outlined in our earlier discussion.

As previously outlined, the effective delivery of miRNA-155 by B cell-derived exosomes has been established. For the delivery of miR-330-3p, researchers may consider the design of a B cell (142) transfected with the V600E mutation. Subsequently, lipid transfection or electroporation can be employed to introduce the miR-330-3p construct into B cells. In addition, the design of a short hairpin RNA (shRNA) or small interfering RNA (siRNA) targeting TPX2 is essential. These molecules should be transfected into B cells along with miR-330-3p for culture, followed by the isolation of exosomes. To enhance the yield of exosomes, researchers can employ additional stimuli, such as TLR3, TLR7, TLR9, and other motifs discussed earlier.It is crucial to emphasize that researchers must continually optimize and thoroughly validate the experimental design at each stage to ensure the exosomes effectively downregulate TPX2 and inhibit melanoma growth. It should be noted that miR-330-3p and TPX2 are merely two components of a complex regulatory network, and their specific roles and effects on tumor growth are contingent upon various factors in the experimental context. Furthermore, although the production of exosomes with multiple engineered cargoes is theoretically feasible, it may induce cellular stress or potentially cytotoxic effects, thereby posing challenges for manipulation.

The study of B-cell-derived exosomes for cancer therapy presents a number of challenges, including inadequate comprehension of their interactions within the complex milieu of TME and insufficient clinical investigations to verify their functionality and potential side effects. We anticipate that with increasing research into B-cell-derived exosomes, their functions and the mechanisms underlying their biological components will soon become clearer.

## Author contributions

JX, FY and GT conceived the study. JX, HC and GY drafted the manuscript. JX, SZ, JZ, LT and ZX performed the literature search and collected the data. ZX, FY and GT helped with the final revision of this manuscript. All authors contributed to the article and approved the submitted version.

## Supplementary material

Supplementary Figure 1 There can be many types of B cells (plasma cells, memory B cells, breg, TIL-B). In their respective ways, they regulate the tumor microenvironment and affect tumor growth.

## References

[1] Sung H Ferlay J Siegel RL Laversanne M Soerjomataram I Jemal A. Global cancer statistics 2020: GLOBOCAN estimates of incidence and mortality worldwide for 36 cancers in 185 countries. CA Cancer J Clin (2021) 71:209–49. doi: 10.3322/caac.21660 33538338

[2] Paget S. The distribution of secondary growths in cancer of the breast. 1889. Cancer Metastasis Rev (1989) 8:98–101. 2673568

[3] Chen F Zhuang X Lin L Yu P Wang Y Shi Y. New horizons in tumor microenvironment biology: challenges and opportunities. BMC Med (2015) 13:45. doi: 10.1186/s12916-015-0278-7 25857315 PMC4350882

[4] Bejarano L Jordāo MJC Joyce JA. Therapeutic targeting of the tumor microenvironment. Cancer Discov (2021) 11:933–59. doi: 10.1158/2159-8290.CD-20-1808 33811125

[5] Xia L Oyang L Lin J Tan S Han Y Wu N. The cancer metabolic reprogramming and immune response. Mol Cancer (2021) 20:28. doi: 10.1186/s12943-021-01316-8 33546704 PMC7863491

[6] Dai J Su Y Zhong S Cong L Liu B Yang J. Exosomes: key players in cancer and potential therapeutic strategy. Signal Transduct Target Ther (2020) 5:145. doi: 10.1038/s41392-020-00261-0 32759948 PMC7406508

[7] Qin SY Zhang AQ Cheng SX Rong L Zhang XZ. Drug self-delivery systems for cancer therapy. Biomaterials (2017) 112:234–47. doi: 10.1016/j.biomaterials.2016.10.016 27768976

[8] Wei G Wang Y Yang G Wang Y Ju R. Recent progress in nanomedicine for enhanced cancer chemotherapy. Theranostics (2021) 11:6370–92. doi: 10.7150/thno.57828 PMC8120226 33995663

[9] Ke X Shen L. Molecular targeted therapy of cancer: the progress and future prospect. Front Lab Med (2017) 1:69–75. doi: 10.1016/j.flm.2017.06.001

[10] Choi H Choi Y Yim HY Mirzaaghasi A Yoo JK Choi C. Biodistribution of exosomes and engineering strategies for targeted delivery of therapeutic exosomes. Tissue Eng Regener Med (2021) 18:499–511. doi: 10.1007/s13770-021-00361-0 PMC8325750 34260047

[11] Zhang X Zhang H Gu J Zhang J Shi H Qian H. Engineered extracellular vesicles for cancer therapy. Adv Mater (2021) 33:e2005709. doi: 10.1002/adma.202005709 33644908

[12] Yang D Zhang W Zhang H Zhang F Chen L Ma L. Progress, opportunity, and perspective on exosome isolation - efforts for efficient exosome-based theranostics. Theranostics (2020) 10:3684–707. doi: 10.7150/thno.41580 PMC7069071 32206116

[13] Dieu-Nosjean MC Giraldo NA Kaplon H Germain C Fridman WH Sautès-Fridman C. Tertiary lymphoid structures, drivers of the anti-tumor responses in human cancers. Immunol Rev (2016) 271:260–75. doi: 10.1111/imr.12405 27088920

[14] Deguchi S Tanaka H Suzuki S Natsuki S Mori T Miki Y. Clinical relevance of tertiary lymphoid structures in esophageal squamous cell carcinoma. BMC Cancer (2022) 22:699. doi: 10.1186/s12885-022-09777-w 35751038 PMC9233387

[15] Ruffin AT Cillo AR Tabib T Liu A Onkar S Kunning SR. B cell signatures and tertiary lymphoid structures contribute to outcome in head and neck squamous cell carcinoma. Nat Commun (2021) 12:3349. doi: 10.1038/s41467-021-23355-x 34099645 PMC8184766

[16] Yadav D Puranik N Meshram A Chavda V Lee PC Jin JO. How advanced are cancer immuno-nanotherapeutics? a comprehensive review of the literature. Int J Nanomed (2023) 18:35–48. doi: 10.2147/IJN.S388349 PMC9830082 36636642

[17] Downs-Canner SM Meier J Vincent BG Serody JS. B cell function in the tumor microenvironment. Annu Rev Immunol (2022) 40:169–93. doi: 10.1146/annurev-immunol-101220-015603 35044794

[18] Fridman WH Meylan M Petitprez F Sun CM Italiano A Sautès-Fridman C. B cells and tertiary lymphoid structures as determinants of tumour immune contexture and clinical outcome. Nat Rev Clin Oncol (2022) 19:441–57. doi: 10.1038/s41571-022-00619-z 35365796

[19] Ghamlouch H Ouled-Haddou H Guyart A Regnier A Trudel S Claisse JF. TLR9 ligand (CpG oligodeoxynucleotide) induces CLL b-cells to differentiate into CD20(+) antibody-secreting cells. Front Immunol (2014) 5:292. doi: 10.3389/fimmu.2014.00292 24982661 PMC4058906

[20] LeBien TW Tedder TF. B lymphocytes: how they develop and function. Blood (2008) 112:1570–80. doi: 10.1182/blood-2008-02-078071 PMC2518873 18725575

[21] Sharonov GV Serebrovskaya EO Yuzhakova DV Britanova OV Chudakov DM. B cells, plasma cells and antibody repertoires in the tumour microenvironment. Nat Rev Immunol (2020) 20:294–307. doi: 10.1038/s41577-019-0257-x 31988391

[22] Kim SS Sumner WA Miyauchi S Cohen EEW Califano JA Sharabi AB. Role of b cells in responses to checkpoint blockade immunotherapy and overall survival of cancer patients. Clin Cancer Res (2021) 27:6075–82. doi: 10.1158/1078-0432.CCR-21-0697 PMC8976464 34230025

[23] Palm AE Henry C. Remembrance of things past: long-term b cell memory after infection and vaccination. Front Immunol (2019) 10:1787. doi: 10.3389/fimmu.2019.01787 31417562 PMC6685390

[24] Cencioni MT Mattoscio M Magliozzi R Bar-Or A Muraro PA. B cells in multiple sclerosis - from targeted depletion to immune reconstitution therapies. Nat Rev Neurol (2021) 17:399–414. doi: 10.1038/s41582-021-00498-5 34075251

[25] Rousset F Peyrol S Garcia E Vezzio N Andujar M Grimaud JA. Long-term cultured CD40-activated b lymphocytes differentiate into plasma cells in response to IL-10 but not IL-4. Int Immunol (1995) 7:1243–53. doi: 10.1093/intimm/7.8.1243 7495731

[26] Shaul ME Zlotnik A Tidhar E Schwartz A Arpinati L Kaisar-Iluz N. Tumor-associated neutrophils drive b-cell recruitment and their differentiation to plasma cells. Cancer Immunol Res (2021) 9:811–24. doi: 10.1158/2326-6066.CIR-20-0839 33906865

[27] Packard TA Cambier JC. B lymphocyte antigen receptor signaling: initiation, amplification, and regulation. F1000Prime Rep (2013) 5:40. doi: 10.12703/P5-40 24167721 PMC3790562

[28] Vitale G Mion F Pucillo C. Regulatory b cells: evidence, developmental origin and population diversity. Mol Immunol (2010) 48:1–8. doi: 10.1016/j.molimm.2010.09.010 20950861

[29] Peng B Ming Y Yang C. Regulatory b cells: the cutting edge of immune tolerance in kidney transplantation. Cell Death Dis (2018) 9:109. doi: 10.1038/s41419-017-0152-y 29371592 PMC5833552

[30] Rosser EC Mauri C. Regulatory b cells: origin, phenotype, and function. Immunity (2015) 42:607–12. doi: 10.1016/j.immuni.2015.04.005 25902480

[31] Rosser EC Mauri C. The emerging field of regulatory b cell immunometabolism. Cell Metab (2021) 33:1088–97. doi: 10.1016/j.cmet.2021.05.008 34077716

[32] Siliņa K Soltermann A Attar FM Casanova R Uckeley ZM Thut H. Germinal centers determine the prognostic relevance of tertiary lymphoid structures and are impaired by corticosteroids in lung squamous cell carcinoma. Cancer Res (2018) 78:1308–20. doi: 10.1158/0008-5472.CAN-17-1987 29279354

[33] Neyt K Perros F GeurtsvanKessel CH Hammad H Lambrecht BN. Tertiary lymphoid organs in infection and autoimmunity. Trends Immunol (2012) 33:297–305. doi: 10.1016/j.it.2012.04.006 22622061 PMC7106385

[34] Wang SS Liu W Ly D Xu H Qu L Zhang L. Tumor-infiltrating b cells: their role and application in anti-tumor immunity in lung cancer. Cell Mol Immunol (2019) 16:6–18. doi: 10.1038/s41423-018-0027-x 29628498 PMC6318290

[35] Yang C Lee H Pal S Jove V Deng J Zhang W. B cells promote tumor progression *via* STAT3 regulated-angiogenesis. PloS One (2013) 8:e64159. doi: 10.1371/journal.pone.0064159 23734190 PMC3667024

[36] Ammirante M Luo JL Grivennikov S Nedospasov S Karin M. B-cell-derived lymphotoxin promotes castration-resistant prostate cancer. Nature (2010) 464:302–5. doi: 10.1038/nature08782 PMC2866639 20220849

[37] Pavlasova G Mraz M. The regulation and function of CD20: an “enigma” of b-cell biology and targeted therapy. Haematologica (2020) 105:1494–506. doi: 10.3324/haematol.2019.243543 PMC7271567 32482755

[38] Ding C Wang L Marroquin J Yan J. Targeting of antigens to b cells augments antigen-specific T-cell responses and breaks immune tolerance to tumor-associated antigen MUC1. Blood (2008) 112:2817–25. doi: 10.1182/blood-2008-05-157396 PMC2556617 18669871

[39] Pylayeva-Gupta Y Das S Handler JS Hajdu CH Coffre M Koralov SB. IL35-producing b cells promote the development of pancreatic neoplasia. Cancer Discovery (2016) 6:247–55. doi: 10.1158/2159-8290.CD-15-0843 PMC5709038 26715643

[40] He Y Qian H Liu Y Duan L Li Y Shi G. The roles of regulatory b cells in cancer. J Immunol Res (2014) 2014:215471. doi: 10.1155/2014/215471 24991577 PMC4060293

[41] Bodogai M Lee Chang C Wejksza K Lai J Merino M Wersto RP. Anti-CD20 antibody promotes cancer escape *via* enrichment of tumor-evoked regulatory b cells expressing low levels of CD20 and CD137L. Cancer Res (2013) 73:2127–38. doi: 10.1158/0008-5472.CAN-12-4184 PMC3618504 23365136

[42] Groeneveld CS Fontugne J Cabel L Bernard-Pierrot I Radvanyi F Allory Y. Tertiary lymphoid structures marker CXCL13 is associated with better survival for patients with advanced-stage bladder cancer treated with immunotherapy. Eur J Cancer (2021) 148:181–9. doi: 10.1016/j.ejca.2021.01.036 33743486

[43] Petitprez F de Reyniès A Keung EZ Chen TW Sun CM Calderaro J. B cells are associated with survival and immunotherapy response in sarcoma. Nature (2020) 577:556–60. doi: 10.1038/s41586-019-1906-8 31942077

[44] Lauss M Donia M Svane IM Jönsson G. B cells and tertiary lymphoid structures: friends or foes in cancer immunotherapy? Clin Cancer Res (2022) 28:1751–8. doi: 10.1158/1078-0432.CCR-21-1130 PMC9306440 34965949

[45] Lu Y Zhao Q Liao JY Song E Xia Q Pan J. Complement signals determine opposite effects of b cells in chemotherapy-induced immunity. Cell (2020) 180:1081–1097.e1024. doi: 10.1016/j.cell.2020.02.015 32142650

[46] Lutz ER Wu AA Bigelow E Sharma R Mo G Soares K. Immunotherapy converts nonimmunogenic pancreatic tumors into immunogenic foci of immune regulation. Cancer Immunol Res (2014) 2:616–31. doi: 10.1158/2326-6066.CIR-14-0027 PMC4082460 24942756

[47] Tokunaga R Naseem M Lo JH Battaglin F Soni S Puccini A. B cell and b cell-related pathways for novel cancer treatments. Cancer Treat Rev (2019) 73:10–9. doi: 10.1016/j.ctrv.2018.12.001 PMC7505165 30551036

[48] Feng Y Liu L Li J Huang J Xie JH Menard L. Systematic characterization of the tumor microenvironment in Chinese patients with hepatocellular carcinoma highlights intratumoral b cells as a potential immunotherapy target. Oncol Rep (2022) 47 (2):38. doi: 10.3892/or.2021.8249 34958112 PMC8717124

[49] Gurunathan S Kang M-H Jeyaraj M Qasim M Kim J-H. Review of the isolation, characterization, biological function, and multifarious therapeutic approaches of exosomes. Cells (2019) 8:307. doi: 10.3390/cells8040307 30987213 PMC6523673

[50] Yan W Jiang S. Immune cell-derived exosomes in the cancer-immunity cycle. Trends Cancer (2020) 6:506–17. doi: 10.1016/j.trecan.2020.02.013 32460004

[51] Mashouri L Yousefi H Aref AR Ahadi AM Molaei F Alahari SK. Exosomes: composition, biogenesis, and mechanisms in cancer metastasis and drug resistance. Mol Cancer (2019) 18:75. doi: 10.1186/s12943-019-0991-5 30940145 PMC6444571

[52] Samuel M Gabrielsson S. Personalized medicine and back-allogeneic exosomes for cancer immunotherapy. J Intern Med (2021) 289:138–46. doi: 10.1111/joim.12963 31359504

[53] Nam GH Choi Y Kim GB Kim S Kim SA Kim IS. Emerging prospects of exosomes for cancer treatment: from conventional therapy to immunotherapy. Adv Mater (2020) 32:e2002440. doi: 10.1002/adma.202002440 33015883

[54] Huang T Deng CX. Current progresses of exosomes as cancer diagnostic and prognostic biomarkers. Int J Biol Sci (2019) 15:1–11. doi: 10.7150/ijbs.27796 30662342 PMC6329932

[55] Wu M Wang G Hu W Yao Y Yu XF. Emerging roles and therapeutic value of exosomes in cancer metastasis. Mol Cancer (2019) 18:53. doi: 10.1186/s12943-019-0964-8 30925925 PMC6441156

[56] Zhang L Yu D. Exosomes in cancer development, metastasis, and immunity. Biochim Biophys Acta Rev Cancer (2019) 1871:455–68. doi: 10.1016/j.bbcan.2019.04.004 PMC6542596 31047959

[57] Ostrowski M Carmo NB Krumeich S Fanget I Raposo G Savina A. Rab27a and Rab27b control different steps of the exosome secretion pathway. Nat Cell Biol (2010) 12:19–30. doi: 10.1038/ncb2000 19966785

[58] Hsu C Morohashi Y Yoshimura S Manrique-Hoyos N Jung S Lauterbach MA. Regulation of exosome secretion by Rab35 and its GTPase-activating proteins TBC1D10A-c. J Cell Biol (2010) 189:223–32. doi: 10.1083/jcb.200911018 PMC2856897 20404108

[59] Wei D Zhan W Gao Y Huang L Gong R Wang W. RAB31 marks and controls an ESCRT-independent exosome pathway. Cell Res (2021) 31:157–77. doi: 10.1038/s41422-020-00409-1 PMC8027411 32958903

[60] Milane L Singh A Mattheolabakis G Suresh M Amiji MM. Exosome mediated communication within the tumor microenvironment. J Control Release (2015) 219:278–94. doi: 10.1016/j.jconrel.2015.06.029 26143224

[61] Tan S Xia L Yi P Han Y Tang L Pan Q. Exosomal miRNAs in tumor microenvironment. J Exp Clin Cancer Res (2020) 39:67. doi: 10.1186/s13046-020-01570-6 32299469 PMC7164281

[62] Kalluri R LeBleu VS. The biology, function, and biomedical applications of exosomes. Science (2020) 367 (6478):eaau6977. doi: 10.1126/science.aau6977 32029601 PMC7717626

[63] Pegtel DM Gould SJ. Exosomes. Annu Rev Biochem (2019) 88:487–514. doi: 10.1146/annurev-biochem-013118-111902 31220978

[64] Tkach M Théry C. Communication by extracellular vesicles: where we are and where we need to go. Cell (2016) 164:1226–32. doi: 10.1016/j.cell.2016.01.043 26967288

[65] Li J Li S Guo J Li Q Long J Ma C. Natural product micheliolide (MCL) irreversibly activates pyruvate kinase M2 and suppresses leukemia. J Med Chem (2018) 61:4155–64. doi: 10.1021/acs.jmedchem.8b00241 PMC5949721 29641204

[66] Shao Y Shen Y Chen T Xu F Chen X Zheng S. The functions and clinical applications of tumor-derived exosomes. Oncotarget (2016) 7:60736–51. doi: 10.18632/oncotarget.11177 PMC5312416 27517627

[67] Yang K Zhou Q Qiao B Shao B Hu S Wang G. Exosome-derived noncoding RNAs: function, mechanism, and application in tumor angiogenesis. Mol Ther Nucleic Acids (2022) 27:983–97. doi: 10.1016/j.omtn.2022.01.009 PMC8905256 35317280

[68] Näslund TI Gehrmann U Qazi KR Karlsson MC Gabrielsson S. Dendritic cell-derived exosomes need to activate both T and b cells to induce antitumor immunity. J Immunol (2013) 190:2712–9. doi: 10.4049/jimmunol.1203082 23418627

[69] Segura E Nicco C Lombard B Véron P Raposo G Batteux F. ICAM-1 on exosomes from mature dendritic cells is critical for efficient naive T-cell priming. Blood (2005) 106:216–23. doi: 10.1182/blood-2005-01-0220 15790784

[70] Tay NQ Lee DCP Chua YL Prabhu N Gascoigne NRJ Kemeny DM. CD40L expression allows CD8(+) T cells to promote their own expansion and differentiation through dendritic cells. Front Immunol (2017) 8:1484. doi: 10.3389/fimmu.2017.01484 29163545 PMC5672143

[71] Bruno S Deregibus MC Camussi G. The secretome of mesenchymal stromal cells: role of extracellular vesicles in immunomodulation. Immunol Lett (2015) 168:154–8. doi: 10.1016/j.imlet.2015.06.007 26086886

[72] Ye L Zhang Q Cheng Y Chen X Wang G Shi M. Tumor-derived exosomal HMGB1 fosters hepatocellular carcinoma immune evasion by promoting TIM-1(+) regulatory b cell expansion. J Immunother Cancer (2018) 6:145. doi: 10.1186/s40425-018-0451-6 30526680 PMC6288912

[73] Schroeder JC Puntigam L Hofmann L Jeske SS Beccard IJ Doescher J. Circulating exosomes inhibit b cell proliferation and activity. Cancers (Basel) (2020) 12 (8):2110. doi: 10.3390/cancers12082110 32751214 PMC7464446

[74] Yang Z Wang W Zhao L Wang X Gimple RC Xu L. Plasma cells shape the mesenchymal identity of ovarian cancers through transfer of exosome-derived microRNAs. Sci Adv (2021) 7 (9):eabb0737. doi: 10.1126/sciadv.abb0737 PMC7904265 33627414

[75] Zhang F Li R Yang Y Shi C Shen Y Lu C. Specific decrease in b-Cell-Derived extracellular vesicles enhances post-chemotherapeutic CD8(+) T cell responses. Immunity (2019) 50:738–750.e737. doi: 10.1016/j.immuni.2019.01.010 30770248

[76] Papp K Végh P Prechl J Kerekes K Kovács J Csikós G. B lymphocytes and macrophages release cell membrane deposited C3-fragments on exosomes with T cell response-enhancing capacity. Mol Immunol (2008) 45:2343–51. doi: 10.1016/j.molimm.2007.11.021 18192019

[77] Momen-Heravi F Bala S Bukong T Szabo G. Exosome-mediated delivery of functionally active miRNA-155 inhibitor to macrophages. Nanomedicine (2014) 10:1517–27. doi: 10.1016/j.nano.2014.03.014 PMC4180003 24685946

[78] Zhu L Sun HT Wang S Huang SL Zheng Y Wang CQ. Isolation and characterization of exosomes for cancer research. J Hematol Oncol (2020) 13:152. doi: 10.1186/s13045-020-00987-y 33168028 PMC7652679

[79] Ma S Zhou M Xu Y Gu X Zou M Abudushalamu G. Clinical application and detection techniques of liquid biopsy in gastric cancer. Mol Cancer (2023) 22:7. doi: 10.1186/s12943-023-01715-z 36627698 PMC9832643

[80] Nikanjam M Kato S Kurzrock R. Liquid biopsy: current technology and clinical applications. J Hematol Oncol (2022) 15:131. doi: 10.1186/s13045-022-01351-y 36096847 PMC9465933

[81] Filipazzi P Bürdek M Villa A Rivoltini L Huber V. Recent advances on the role of tumor exosomes in immunosuppression and disease progression. Semin Cancer Biol (2012) 22:342–9. doi: 10.1016/j.semcancer.2012.02.005 22369922

[82] Whiteside TL. Immune modulation of T-cell and NK (natural killer) cell activities by TEXs (tumour-derived exosomes). Biochem Soc Trans (2013) 41:245–51. doi: 10.1042/BST20120265 PMC3721347 23356291

[83] Wolfers J Lozier A Raposo G Regnault A Théry C Masurier C. Tumor-derived exosomes are a source of shared tumor rejection antigens for CTL cross-priming. Nat Med (2001) 7:297–303. doi: 10.1038/85438 11231627

[84] Zhang HG Zhuang X Sun D Liu Y Xiang X Grizzle WE. Exosomes and immune surveillance of neoplastic lesions: a review. Biotech Histochem (2012) 87:161–8. doi: 10.3109/10520291003659042 PMC3445025 22216980

[85] Zuo B Zhang Y Zhao K Wu L Qi H Yang R. Universal immunotherapeutic strategy for hepatocellular carcinoma with exosome vaccines that engage adaptive and innate immune responses. J Hematol Oncol (2022) 15:46. doi: 10.1186/s13045-022-01266-8 35488312 PMC9052531

[86] van der Pol E Böing AN Harrison P Sturk A Nieuwland R. Classification, functions, and clinical relevance of extracellular vesicles. Pharmacol Rev (2012) 64:676–705. doi: 10.1124/pr.112.005983 22722893

[87] Mondal J Pillarisetti S Junnuthula V Saha M Hwang SR Park IK. Hybrid exosomes, exosome-like nanovesicles and engineered exosomes for therapeutic applications. J Control Release (2023) 353:1127–49. doi: 10.1016/j.jconrel.2022.12.027 36528193

[88] Liu C Su C. Design strategies and application progress of therapeutic exosomes. Theranostics (2019) 9:1015–28. doi: 10.7150/thno.30853 PMC6401399 30867813

[89] Prajapati S Hinchliffe T Roy V Shah N Jones CN Obaid G. Biomimetic nanotechnology: a natural path forward for tumor-selective and tumor-specific NIR activable photonanomedicines. Pharmaceutics (2021) 13 (6) :786. doi: 10.3390/pharmaceutics13060786 34070233 PMC8225032

[90] Turturici G Tinnirello R Sconzo G Geraci F. Extracellular membrane vesicles as a mechanism of cell-to-cell communication: advantages and disadvantages. Am J Physiol Cell Physiol (2014) 306:C621–633. doi: 10.1152/ajpcell.00228.2013 24452373

[91] Batrakova EV Kim MS. Using exosomes, naturally-equipped nanocarriers, for drug delivery. J Control Release (2015) 219:396–405. doi: 10.1016/j.jconrel.2015.07.030 26241750 PMC4656109

[92] Saari H Lázaro-Ibáñez E Viitala T Vuorimaa-Laukkanen E Siljander P Yliperttula M. Microvesicle- and exosome-mediated drug delivery enhances the cytotoxicity of paclitaxel in autologous prostate cancer cells. J Control Release (2015) 220:727–37. doi: 10.1016/j.jconrel.2015.09.031 26390807

[93] Zhang Y Li L Yu J Zhu D Zhang Y Li X. Microvesicle-mediated delivery of transforming growth factor β1 siRNA for the suppression of tumor growth in mice. Biomaterials (2014) 35:4390–400. doi: 10.1016/j.biomaterials.2014.02.003 24565517

[94] Zhao L Gu C Gan Y Shao L Chen H Zhu H. Exosome-mediated siRNA delivery to suppress postoperative breast cancer metastasis. J Control Release (2020) 318:1–15. doi: 10.1016/j.jconrel.2019.12.005 31830541

[95] Lockman PR Mittapalli RK Taskar KS Rudraraju V Gril B Bohn KA. Heterogeneous blood-tumor barrier permeability determines drug efficacy in experimental brain metastases of breast cancer. Clin Cancer Res (2010) 16:5664–78. doi: 10.1158/1078-0432.CCR-10-1564 PMC2999649 20829328

[96] Yang T Martin P Fogarty B Brown A Schurman K Phipps R. Exosome delivered anticancer drugs across the blood-brain barrier for brain cancer therapy in danio rerio. Pharm Res (2015) 32:2003–14. doi: 10.1007/s11095-014-1593-y PMC4520542 25609010

[97] Casey TM Eneman J Crocker A White J Tessitore J Stanley M. Cancer associated fibroblasts stimulated by transforming growth factor beta1 (TGF-beta 1) increase invasion rate of tumor cells: a population study. Breast Cancer Res Treat (2008) 110:39–49. doi: 10.1007/s10549-007-9684-7 17674196

[98] Fridlender ZG Sun J Kim S Kapoor V Cheng G Ling L. Polarization of tumor-associated neutrophil phenotype by TGF-beta: “N1” versus “N2” TAN. Cancer Cell (2009) 16:183–94. doi: 10.1016/j.ccr.2009.06.017 PMC2754404 19732719

[99] Chen Y Zheng X Wu C. The role of the tumor microenvironment and treatment strategies in colorectal cancer. Front Immunol (2021) 12:792691. doi: 10.3389/fimmu.2021.792691 34925375 PMC8674693

[100] Ren B Cui M Yang G Wang H Feng M You L. Tumor microenvironment participates in metastasis of pancreatic cancer. Mol Cancer (2018) 17:108. doi: 10.1186/s12943-018-0858-1 30060755 PMC6065152

[101] Rong L Li R Li S Luo R. Immunosuppression of breast cancer cells mediated by transforming growth factor-β in exosomes from cancer cells. Oncol Lett (2016) 11:500–4. doi: 10.3892/ol.2015.3841 PMC4727188 26870240

[102] Ricklefs FL Alayo Q Krenzlin H Mahmoud AB Speranza MC Nakashima H. Immune evasion mediated by PD-L1 on glioblastoma-derived extracellular vesicles. Sci Adv (2018) 4:eaar2766. doi: 10.1126/sciadv.aar2766 29532035 PMC5842038

[103] Theodoraki MN Yerneni SS Hoffmann TK Gooding WE Whiteside TL. Clinical significance of PD-L1(+) exosomes in plasma of head and neck cancer patients. Clin Cancer Res (2018) 24:896–905. doi: 10.1158/1078-0432.CCR-17-2664 29233903 PMC6126905

[104] Turiello R Capone M Morretta E Monti MC Madonna G Azzaro R. Exosomal CD73 from serum of patients with melanoma suppresses lymphocyte functions and is associated with therapy resistance to anti-PD-1 agents. J Immunother Cancer (2022) 10:e004043. doi: 10.1136/jitc-2021-004043 35273100 PMC8915288

[105] Theodoraki MN Yerneni S Gooding WE Ohr J Clump DA Bauman JE. Circulating exosomes measure responses to therapy in head and neck cancer patients treated with cetuximab, ipilimumab, and IMRT. Oncoimmunology (2019) 8:1593805. doi: 10.1080/2162402X.2019.1593805 31143513 PMC6527269

[106] Raposo G Nijman HW Stoorvogel W Liejendekker R Harding CV Melief CJ. B lymphocytes secrete antigen-presenting vesicles. J Exp Med (1996) 183:1161–72. doi: 10.1084/jem.183.3.1161 PMC2192324 8642258

[107] Wubbolts R Leckie RS Veenhuizen PT Schwarzmann G Möbius W Hoernschemeyer J. Proteomic and biochemical analyses of human b cell-derived exosomes. Potential implications their Funct multivesicular body formation. J Biol Chem (2003) 278:10963–72. doi: 10.1074/jbc.M207550200 12519789

[108] Clayton A Court J Navabi H Adams M Mason MD Hobot JA. Analysis of antigen presenting cell derived exosomes, based on immuno-magnetic isolation and flow cytometry. J Immunol Methods (2001) 247:163–74. doi: 10.1016/S0022-1759(00)00321-5 11150547

[109] Saunderson SC Schuberth PC Dunn AC Miller L Hock BD MacKay PA. Induction of exosome release in primary b cells stimulated *via* CD40 and the IL-4 receptor. J Immunol (2008) 180:8146–52. doi: 10.4049/jimmunol.180.12.8146 18523279

[110] Ismail N Wang Y Dakhlallah D Moldovan L Agarwal K Batte K. Macrophage microvesicles induce macrophage differentiation and miR-223 transfer. Blood (2013) 121:984–95. doi: 10.1182/blood-2011-08-374793 PMC3567345 23144169

[111] Arita S Baba E Shibata Y Niiro H Shimoda S Isobe T. B cell activation regulates exosomal HLA production. Eur J Immunol (2008) 38:1423–34. doi: 10.1002/eji.200737694 18425730

[112] Calvo V Izquierdo M. Inducible polarized secretion of exosomes in T and b lymphocytes. Int J Mol Sci (2020) 21 (7) :2631. doi: 10.3390/ijms21072631 32290050 PMC7177964

[113] Penninger JM Irie-Sasaki J Sasaki T Oliveira-dos-Santos AJ. CD45: new jobs for an old acquaintance. Nat Immunol (2001) 2:389–96. doi: 10.1038/87687 11323691

[114] Hermiston ML Xu Z Weiss A. CD45: a critical regulator of signaling thresholds in immune cells. Annu Rev Immunol (2003) 21:107–37. doi: 10.1146/annurev.immunol.21.120601.140946 12414720

[115] Blanchard N Lankar D Faure F Regnault A Dumont C Raposo G. TCR activation of human T cells induces the production of exosomes bearing the TCR/CD3/zeta complex. J Immunol (2002) 168:3235–41. doi: 10.4049/jimmunol.168.7.3235 11907077

[116] Richmond A Nf-kappa B. Chemokine gene transcription and tumour growth. Nat Rev Immunol (2002) 2:664–74. doi: 10.1038/nri887 PMC2668257 12209135

[117] Lindenbergh MFS Stoorvogel W. Antigen presentation by extracellular vesicles from professional antigen-presenting cells. Annu Rev Immunol (2018) 36:435–59. doi: 10.1146/annurev-immunol-041015-055700 29400984

[118] Buschow SI van Balkom BW Aalberts M Heck AJ Wauben M Stoorvogel W. MHC class II-associated proteins in b-cell exosomes and potential functional implications for exosome biogenesis. Immunol Cell Biol (2010) 88:851–6. doi: 10.1038/icb.2010.64 20458337

[119] Léveillé C AL-D R Mourad W. CD20 is physically and functionally coupled to MHC class II and CD40 on human b cell lines. Eur J Immunol (1999) 29:65–74. doi: 10.1002/(SICI)1521-4141(199901)29:01<65::AID-IMMU65>3.0.CO;2-E 9933087

[120] Munoz-Erazo L Rhodes JL Marion VC Kemp RA. Tertiary lymphoid structures in cancer - considerations for patient prognosis. Cell Mol Immunol (2020) 17:570–5. doi: 10.1038/s41423-020-0457-0 PMC7264315 32415259

[121] Polyak MJ Li H Shariat N Deans JP. CD20 homo-oligomers physically associate with the b cell antigen receptor. dissociation upon receptor engagement and recruitment of phosphoproteins and calmodulin-binding proteins. J Biol Chem (2008) 283:18545–52. doi: 10.1074/jbc.M800784200 18474602

[122] Bubien JK Zhou LJ Bell PD Frizzell RA Tedder TF. Transfection of the CD20 cell surface molecule into ectopic cell types generates a Ca2+ conductance found constitutively in b lymphocytes. J Cell Biol (1993) 121:1121–32. doi: 10.1083/jcb.121.5.1121 PMC2119683 7684739

[123] Sato Y Shimoda M Sota Y Miyake T Tanei T Kagara N. Enhanced humoral immunity in breast cancer patients with high serum concentration of anti-HER2 autoantibody. Cancer Med (2021) 10:1418–30. doi: 10.1002/cam4.3742 PMC7926031 33506656

[124] Meshcheryakova A Tamandl D Bajna E Stift J Mittlboeck M Svoboda M. B cells and ectopic follicular structures: novel players in anti-tumor programming with prognostic power for patients with metastatic colorectal cancer. PloS One (2014) 9:e99008. doi: 10.1371/journal.pone.0099008 24905750 PMC4048213

[125] Muntasell A Berger AC Roche PA. T Cell-induced secretion of MHC class II-peptide complexes on b cell exosomes. EMBO J (2007) 26:4263–72. doi: 10.1038/sj.emboj.7601842 PMC2230838 17805347

[126] Knight AM. Regulated release of b cell-derived exosomes: do differences in exosome release provide insight into different APC function for b cells and DC? Eur J Immunol (2008) 38:1186–9. doi: 10.1002/eji.200838374 18425725

[127] Liao TL Hsieh SL Chen YM Chen HH Liu HJ Lee HC. Rituximab may cause increased hepatitis c virus viremia in rheumatoid arthritis patients through declining exosomal MicroRNA-155. Arthritis Rheumatol (2018) 70:1209–19. doi: 10.1002/art.40495 29575671

[128] Yao Y Zuo J Wei Y. Targeting of TRX2 by miR-330-3p in melanoma inhibits proliferation. BioMed Pharmacother (2018) 107:1020–9. doi: 10.1016/j.biopha.2018.08.058 30257313

[129] Ma Y Lin D Sun W Xiao T Yuan J Han N. Expression of targeting protein for xklp2 associated with both malignant transformation of respiratory epithelium and progression of squamous cell lung cancer. Clin Cancer Res (2006) 12:1121–7. doi: 10.1158/1078-0432.CCR-05-1766 16489064

[130] Arcangeli ML Frontera V Bardin F Thomassin J Chetaille B Adams S. The junctional adhesion molecule-b regulates JAM-c-dependent melanoma cell metastasis. FEBS Lett (2012) 586:4046–51. doi: 10.1016/j.febslet.2012.10.005 23068611

[131] Costa A Kieffer Y Scholer-Dahirel A Pelon F Bourachot B Cardon M. Fibroblast heterogeneity and immunosuppressive environment in human breast cancer. Cancer Cell (2018) 33:463–479.e410. doi: 10.1016/j.ccell.2018.01.011 29455927

[132] Denzer K van Eijk M Kleijmeer MJ Jakobson E de Groot C Geuze HJ. Follicular dendritic cells carry MHC class II-expressing microvesicles at their surface. J Immunol (2000) 165:1259–65. doi: 10.4049/jimmunol.165.3.1259 10903724

[133] Koopman G Keehnen RM Lindhout E Newman W Shimizu Y van Seventer GA. Adhesion through the LFA-1 (CD11a/CD18)-ICAM-1 (CD54) and the VLA-4 (CD49d)-VCAM-1 (CD106) pathways prevents apoptosis of germinal center b cells. J Immunol (1994) 152:3760–7. doi: 10.4049/jimmunol.152.8.3760 7511659

[134] Gray D Kosco M Stockinger B. Novel pathways of antigen presentation for the maintenance of memory. Int Immunol (1991) 3:141–8. doi: 10.1093/intimm/3.2.141 2025614

[135] Vermi W Lonardi S Bosisio D Uguccioni M Danelon G Pileri S. Identification of CXCL13 as a new marker for follicular dendritic cell sarcoma. J Pathol (2008) 216:356–64. doi: 10.1002/path.2420 18792075

[136] Clayton A Turkes A Dewitt S Steadman R Mason MD Hallett MB. Adhesion and signaling by b cell-derived exosomes: the role of integrins. FASEB J (2004) 18:977–9. doi: 10.1096/fj.03-1094fje 15059973

[137] Saunderson SC Dunn AC Crocker PR McLellan AD. CD169 mediates the capture of exosomes in spleen and lymph node. Blood (2014) 123:208–16. doi: 10.1182/blood-2013-03-489732 PMC3888287 24255917

[138] Berney C Herren S Power CA Gordon S Martinez-Pomares L Kosco-Vilbois MH. A member of the dendritic cell family that enters b cell follicles and stimulates primary antibody responses identified by a mannose receptor fusion protein. J Exp Med (1999) 190:851–60. doi: 10.1084/jem.190.6.851 PMC2195630 10499923

[139] Martínez-Pomares L Kosco-Vilbois M Darley E Tree P Herren S Bonnefoy JY. Fc chimeric protein containing the cysteine-rich domain of the murine mannose receptor binds to macrophages from splenic marginal zone and lymph node subcapsular sinus and to germinal centers. J Exp Med (1996) 184:1927–37. doi: 10.1084/jem.184.5.1927 PMC2192889 8920880

[140] Martinez-Pomares L Gordon S. CD169+ macrophages at the crossroads of antigen presentation. Trends Immunol (2012) 33:66–70. doi: 10.1016/j.it.2011.11.001 22192781

[141] Qazi KR Gehrmann U Domange Jordö E Karlsson MC Gabrielsson S. Antigen-loaded exosomes alone induce Th1-type memory through a b-cell-dependent mechanism. Blood (2009) 113:2673–83. doi: 10.1182/blood-2008-04-153536 19176319

[142] Menzer C Menzies AM Carlino MS Reijers I Groen EJ Eigentler T. Targeted therapy in advanced melanoma with rare BRAF mutations. J Clin Oncol (2019) 37:3142–51. doi: 10.1200/JCO.19.00489 PMC10448865 31580757

