# Supplementary figures and images for: Revolutionizing anti-tumor therapy: unleashing the potential of B cell-derived exosomes
Source: Front Immunol. 2023 Jun 5;14:1188760. doi: 10.3389/fimmu.2023.1188760 (PMC10277631; doi:10.3389/fimmu.2023.1188760)

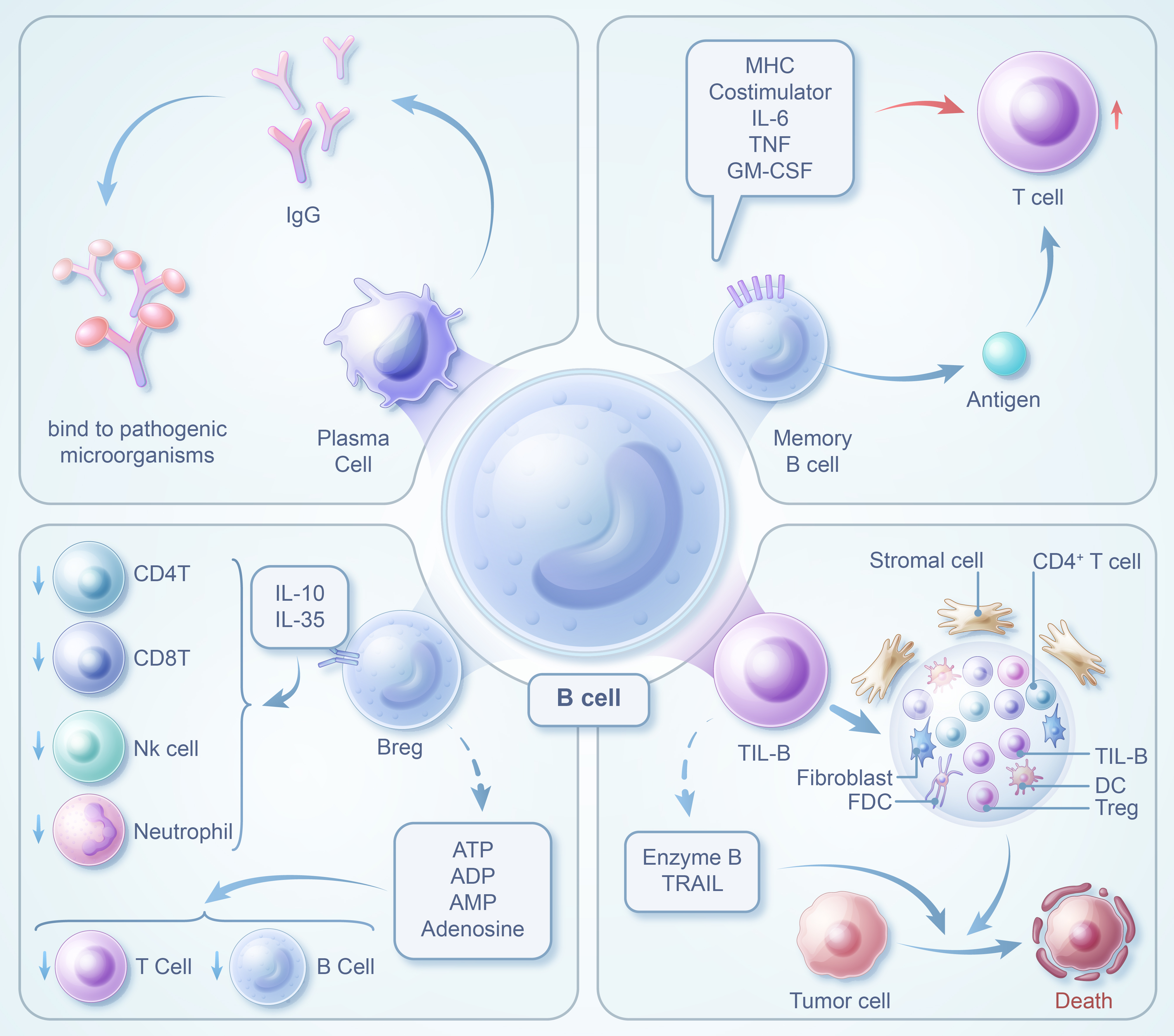

Supplement: Supplementary Figure 1 — There can be many types of B cells (plasma cells, memory B cells, breg, TIL-B). In their respective ways, they regulate the tumor microenvironment and affect tumor growth. [file Image_1.jpeg]
